# Hypertension and Blood Pressure Dysregulation in Cancer Survivors: Mechanisms, Management, and Future Directions

**DOI:** 10.1007/s11906-026-01394-3

**Published:** 2026-11-10

**Authors:** Lauren Daniel, W. Gregory Hundley

**Affiliations:** VCU Pauley Heart Center, Division of Cardiology, Department of Internal Medicine, Virginia Commonwealth University, P.O. Box 980335, 1200 East Broad Street, Richmond, VA 23298 USA

**Keywords:** Cardio-oncology, Hypertension, Cancer therapy-related hypertension, Autonomic dysfunction, Blood pressure lability, Cancer survivorship

## Abstract

**Purpose of Review:**

This review examines the epidemiology, mechanisms, and management of hypertension in patients with cancer, emphasizing treatment-related hypertension, autonomic dysfunction, blood pressure lability, and evolving blood pressure targets in cardio-oncology.

**Recent Findings:**

Among cancer survivors both during and after their cancer treatment, hypertension is highly prevalent and is associated with increased cardiovascular morbidity and mortality. Vascular endothelial growth factor (VEGF) inhibitors are one of the most common causes of cancer treatment-induced hypertension, although other cancer treatments also contribute. Emerging evidence highlights the coexistence of orthostatic hypotension and hypertension, likely related to autonomic dysfunction, arterial stiffening, baroreflex impairment, and endothelial dysfunction related to cancer and its treatment. Medical management, intensive blood pressure control, and cardio-oncology rehabilitation may improve blood pressure control while mitigating adverse outcomes.

**Summary:**

Current hypertension management in cancer survivors largely follows general population guidelines despite the unique pathophysiology of cancer and its therapies. Future research should establish cancer-specific blood pressure targets, optimize antihypertensive strategies, and further investigate management of blood pressure lability, autonomic dysfunction, and cardio-oncology rehabilitation.

## Introduction

Practitioners in cardio-oncology, an evolving field focused on the treatment and prevention of cardiovascular disease among those with or treated for cancer, recognize hypertension (HTN) as an important modifiable risk factor in need of treatment to reduce the occurrence of adverse events [1, 2]. This review focuses on epidemiology and etiologies of HTN in patients with cancer, cancer therapies that promote HTN, autonomic dysfunction and blood pressure lability, medical management of HTN, and blood pressure goals in those with or treated for cancer.

## Hypertension in Cancer Patients

### Cardiovascular Events Related to Hypertension

One factor that influences the risk of developing HTN during and after cancer treatment is the presence of pre-existing HTN before initiating therapy. HTN at the time of cancer diagnosis is associated with increased risk of both all-cause mortality and cancer-specific mortality compared to individuals without HTN [3].

Additionally, HTN severity impacts the risk of heart failure progression and left ventricular decline in patients with cancer. Higher blood pressure (BP) is associated with an increased risk for heart failure progression and other cardiovascular events, with a study showing that hazard ratios for heart failure were 1.15 (95% CI, 0.93 to 1.44) for elevated BP, 1.24 (95% CI, 1.03 to 1.49) for stage 1 HTN, and 1.99 (95% CI, 1.63 to 2.43) for stage 2 HTN in patients with a history of breast, colorectal, or stomach cancer [4]. Furthermore, among breast cancer survivors, a designation assigned at the time of cancer diagnosis, those with stage 2 HTN experience left ventricular ejection fraction declines compared to those with normal BP across the first three months of potentially cardiotoxic chemotherapy [5].

### Prevalence of Hypertension in Cancer Survivors

Hypertension is the most common cardiovascular comorbidity experienced by cancer survivors, with HTN seen in up to 38% of cancer survivors [2]. Furthermore, HTN can be highly prevalent in cancer survivors long after their diagnosis and subsequent treatment [1]. According to the St. Jude Lifetime Cohort data, survivors of childhood cancer have a higher prevalence of HTN (70% developing HTN by age 50) compared to the general population [6]. These findings highlight HTN as a persistent and clinically important cardiovascular complication among cancer survivors.

### Inequities in Oncology Patients: Race and Social Determinants of Health

Social factors including race and social determinants of health are also associated with HTN among cancer survivors. In an oncology population, Black breast cancer survivors had a 30% higher prevalence of HTN (PR = 1.30 [95% CI, 1.11–1.52]) than white breast cancer survivors [7]. In cancer patients, adverse social determinants of health are also found to be associated with an increased risk of uncontrolled HTN (SBP > 140 and/or DBP > 90) [8], and cancer survivors with both adverse socioeconomic status and hypertension have a 3.4-fold higher risk of CVD mortality compared to patients of higher socioeconomic status without hypertension. Low socioeconomic status interacts synergistically with hypertension in cancer survivors to increase the risk of cardiovascular mortality [9]. These findings suggest that patients from marginalized communities may be more likely to experience HTN and subsequent adverse cardiovascular outcomes during cancer treatment and survivorship. This highlights the need for increased screening and subsequent treatment for HTN in these populations, as well as additional research to further investigate potential etiologies behind these findings.

### Home Blood Pressure Monitoring

Furthermore, home blood pressure monitoring may be advantageous due to the high prevalence of white-coat hypertension in cancer patients. Although white-coat hypertension in cancer patients is recognized in clinical practice, data on its prevalence is limited. In a study comparing BP measurements by physicians versus nurses in breast cancer patients, white-coat hypertension was found in 59% of patients [10]. The American Heart Association and Heart Failure Society of America recognize the high prevalence of white-coat hypertension in cancer patients and encourage home blood pressure monitoring [11, 12].

### Bidirectional Relationships and Shared Risk Factors Between HTN and Cancer

It is well established that cancer treatments may often cause HTN, but emerging research also suggests that there may be a bidirectional relationship between HTN and cancer [11]. This is predominantly evident in renal cell carcinoma, where systolic and diastolic blood pressure are both positively associated with risk of kidney cancer [13]. Positive relationships have also been established between HTN and colorectal and breast cancer [13]. Additional research is needed to confirm these findings, but it is possible that HTN may be a risk factor for the development of some cancers.

Furthermore, some factors increase the risk of both cancer and hypertension, which could exacerbate their frequent concurrence. Tobacco use, diabetes, increased body mass index, inflammation, and oxidative stress are heavily implicated in both conditions [11]. Cancer treatments are heavily associated with the development of HTN in oncology patients, but it is likely that these shared risk factors could play an additional role in the pathophysiology of these concurrent disease processes.

## Cancer Therapies Shown to Promote Hypertension

Several cancer therapies promote hypertension (Table 1). These therapies differ in incidence, mechanism, and magnitude of blood pressure elevation. Vascular endothelial growth factor (VEGF) inhibitors are associated with the highest incidence of HTN (Table 1). These drugs function as anti-cancer agents by inhibiting VEGF-mediated tumor angiogenesis, thereby reducing tumor blood supply. VEGF inhibitors promote HTN through reduced nitric oxide bioavailability, increased endothelin-1 production, capillary rarefaction, oxidative stress, vascular inflammation, and impaired renal natriuresis [11, 14, 15]. These mechanisms collectively promote vascular dysfunction and hypertension.

**Table 1:** Hypertension Associated with Cancer Therapies. Incidence, magnitude of blood pressure elevation, and proposed mechanisms of treatment-associated hypertension are summarized for select classes of anticancer therapies

Drug Class	Malignancies Treated	Primary Mechanism of HTN	Incidence of HTN	Magnitude of BP elevation
VEGF Monoclonal Antibody	Renal, hepatocellular, thyroid, gastrointestinal stromal (GIST)	Decreased NO production via PI3K-Akt/eNOS; ↑ endothelin−1; capillary rarefaction; impaired renal natriuresis [11, 14]	20–90% [15, 16]	SBP: +21 mmHg DBP: +15 mmHg [17]
VEGFR- Directed TKIs	Renal, hepatocellular, thyroid, gastrointestinal stromal (GIST)	Same as VEGF monoclonal antibody, but also with increased endothelin−1 production and inhibited PDGFB-R signaling [14]	5–80% [18]	SBP: +8.5 mmHg DBP: 6.7 mmHg [19]
ABL- Directed TKIs	CML, BCR-ABL positive AML, ALL	Reduced endothelial cell c-Abl signaling and cell survival Reduced VEGF-R2 signaling with reduction in endothelial function, survival, and proliferation [14]	33% [20]	17% develop grade 3 HTN (SBP > 180 mmHg) [20]
BTK Inhibitors	Chronic lymphocytic leukemia (CLL), Mantle cell lymphoma	Unclear, proposed mechanism related to decrease in heat shock protein 70 downstream of the toll-like receptor-BTK signaling pathway and inhibition of PI3K- dependent NO production [21]	Up to 72% [22]	SBP: +5.2 mmHg [22]
Proteasome Inhibitors	Multiple myeloma, AL amyloidosis, Waldenström’s macroglobulinemia	Vascular oxidative stress Endothelial dysfunction and injury [23]	9%−27% [23]	SBP: +10 mmHg DBP + 3.3 mmHg [24]
Anthracyclines	Breast cancer, lymphoma, leukemia sarcoma, ovarian, bladder	Vascular injury, vasoconstriction through GPCRs and the MEK/ERK1/2 pathway, ET_A_, 5-HT_1B_, and TP receptor mediated vasoconstriction [25] Increased arterial stiffness [26]	Not well established	Not well established

*Abbreviations*: *ABL* Abelson tyrosine kinase, *ALL* Acute lymphoblastic leukemia, *AML* Acute myeloid leukemia, *BP* Blood pressure, *BTK* Bruton tyrosine kinase, *CML* Chronic myeloid leukemia, *DBP* Diastolic blood pressure, *eNOS* Endothelial nitric oxide synthase, *ET* Endothelin, *GIST* Gastrointestinal stromal tumor, *HTN* Hypertension, *NO* Nitric oxide, *PDGFB-R* Platelet-derived growth factor beta receptor, *PI3K* Phosphoinositide 3-kinase, *SBP* Systolic blood pressure, *TKI* Tyrosine kinase inhibitor, *TP* Thromboxane/prostanoid, *VEGF* Vascular endothelial growth factor, *VEGFR* Vascular endothelial growth factor receptor

Many other therapies are also associated with HTN, including Tyrosine Kinase Inhibitors (TKIs), Bruton Tyrosine Kinase (BTK) inhibitors, proteasome inhibitors, and anthracycline chemotherapies, as shown in Table 1. VEGFR-directed TKIs share similar mechanisms to VEGF-directed monoclonal antibodies, but they also exhibit increased endothelin-1 production and inhibition of platelet-derived growth factor signaling [14]. ABL-directed TKIs primarily promote HTN through endothelial cell dysfunction, and BTK inhibitors are proposed to cause HTN through a decrease in heat shock protein 70 and inhibition of PI3K-dependent NO production [14, 21, 27]. Proteasome inhibitors can cause vascular oxidative stress and endothelial dysfunction [14].

Anthracycline chemotherapies are shown to cause vascular injury and vasoconstriction, but they also induce hypertension by causing increased arterial stiffening [25, 26]. This stiffening is driven by impaired endothelial function, decreased nitric oxide, oxidative stress [28, 29], cellular senescence and the senescence-associated secretory phenotype axis [30], and increased aortic fibrosis and collagen deposition [31].

## Medical Management of Hypertension in Patients with Cancer

As discussed previously, cancer therapies are associated with changes in blood pressure that may be labile, unpredictable, and therefore potentially difficult to monitor. Recent guidelines encourage baseline BP measurement before initiating prohypertensive cancer therapies, and at least weekly monitoring during the first four to eight weeks of treatment [11, 32]. These guidelines also recommend close blood pressure monitoring upon discontinuation of potentially prohypertensive cancer therapies, as rebound hypotension can occur upon their discontinuation [11, 32].

### When to Initiate Antihypertensives

Currently, the guidelines on when to initiate antihypertensives in patients with cancer are the same as recommended for the general population. It is recommended to initiate pharmacotherapy at stage 1 HTN (≥ 130/80 mmHg) for patients with ASCVD 10-year risk ≥ 10% or comorbidities such as chronic kidney disease or diabetes. For patients without these risk factors, guidelines recommend initiating treatment at stage 2 HTN (≥ 140/90 mmHg) [11]. However, it is important to consider that many randomized controlled trials on HTN have not included cancer patients, so additional research on BP goals in cancer patients is needed.

### Which Antihypertensives are Indicated in Patients with Cancer?

For general cancer patients, the first-line agents currently recommended are angiotensin-converting enzyme (ACE) inhibitors or angiotensin II receptor blockers (ARBs), dihydropyridine calcium channel blockers (CCB), or thiazide/thiazide-like diuretics [11]. However, caution is warranted when initiating diuretics, as cancer patients are at a high risk for volume depletion and autonomic dysfunction, as discussed in the Figure.

Additional patient-specific risk factors and comorbidities further influence the selection of antihypertensive therapy. ACE inhibitors or ARBs are preferred in patients with proteinuria [33]. In those with a high risk of volume depletion, diuretics should be avoided [34]. In patients with resistant hypertension, current recommendations support mineralocorticoid receptor antagonists [35]. A special consideration is that beta-blockers should not be first-line unless there is a specific indication, such as atrial fibrillation, recent myocardial infarction, or heart failure with reduced ejection fraction [33, 35]. Lifestyle modifications should be considered in all patients, including sodium restriction, limiting nonsteroidal anti-inflammatory drug use, limiting caffeine and alcohol, and increasing physical activity [33, 35]. Lastly, some other therapies used adjunctively to cancer therapies can be prohypertensive, such as erythropoietin-stimulating agents and corticosteroids. It is important to manage these as well in cancer patients with elevated BP [11].

### Hypertension Management in Patients on VEGF Inhibitors

The reported incidence of HTN with VEGF inhibitors ranges from 5% to 90%, depending on the specific agent and patient population. As the cancer therapy most prevalently associated with HTN, there are some treatment specific guidelines for patients on VEGF inhibitors, as shown in Table 2. Notably, anti-VEGF agents should be withheld if systolic blood pressure (SBP) is greater than or equal to 180 mmHg or if diastolic blood pressure (DBP) is greater than or equal to 110 mmHg. These agents can be re-started once BP is less than or equal to 160/110 mmHg on antihypertensive therapy [11, 12, 32].

**Table 2:** Medical management of hypertension in patients receiving VEGF inhibitors. Suggested approach to blood pressure monitoring and antihypertensive therapy in patients receiving VEGF inhibitors

Blood Pressure, mmHg	Guidelines	First-Line Antihypertensive	Second-Line Antihypertensive, if BP not controlled	Third-Line Antihypertensives, if BP not controlled
Target BP < 130/80	Continue BP monitoring	No additional therapy indicated	No additional therapy indicated	No additional therapy indicated
BP ≥ 130/80 but < 160/100	Start Antihypertensive Therapy	ACEi/ARB	Add Dihydropyridine CCBs	Resistant HTN, consider adding Beta-Blockers, Spironolactone, or Nitrates + Hydralazine
BP ≥ 160/100 but < 180/110	Start Antihypertensive therapy	ACEi/ARB and Dihydropyridine CCBs	Resistant HTN, consider adding Beta-Blockers, Spironolactone, or Nitrates + Hydralazine	
BP ≥ 180/110	Hold VEGF Inhibitor until BP < 160/100 and initiate antihypertensive therapy	ACEi/ARB and Dihydropyridine CCBs	Resistant HTN, consider adding Beta-Blockers, Spironolactone, or Nitrates and Hydralazine [31]	

*Abbreviations*: *ACEi* Angiotensin-converting enzyme inhibitor, *ARB* Angiotensin II receptor blocker, *BP* Blood pressure, *CCB* Calcium channel blocker, *HTN* Hypertension, *VEGF* Vascular endothelial growth factor

### “HTN as a Biomarker” Paradox

VEGF inhibitors are often used to treat renal cell carcinoma, which is also commonly associated with HTN. However, there is some controversy over whether this HTN seen during therapy is harmful or potentially beneficial to survival. Studies have shown that in treatment of renal cell carcinoma with VEGF inhibitors, blood pressure elevation was associated with an improved median overall survival compared with no blood pressure elevation (45.4 versus 20.3 months, respectively, *p* = 0.003) [27]. For HTN refractory to first-line agents, long-acting nitrates may be used for improved BP control. However, because nitric oxide plays an important role in VEGF-mediated angiogenesis, there is theoretical concern that long-acting nitrates could interfere with the antiangiogenic mechanism of VEGF inhibitors [36, 37]. However, there are still no guidelines that recommend withholding or reducing antihypertensive medications to promote efficacy of VEGF inhibitors.

### Considerations for Choosing Antihypertensives in Patients Receiving VEGF Inhibitors

Recent treatment guidelines from the American College of Cardiology for patients on VEGF inhibitors indicates ACE inhibitors/ARBs and dihydropyridine CCBs as the first-line treatment for patients receiving VEGF inhibitors (Table 2).

The suggestion for ACE inhibitors/ARBs as first-line agents is because of the theoretical possibility of limiting progression to heart failure, although a recent study found that adding enalapril to standard chemotherapy did not prevent cardiotoxicity in patients receiving high-dose anthracycline chemotherapy [38]. Dihydropyridine CCBs are recommended specifically over non-dihydropyridine CCBs because vascular dysfunction is a key component of the pathophysiology of VEGF inhibitor-induced HTN. Therefore, the vasodilatory effects of smooth muscle relaxation may be particularly beneficial in this setting [27]. Furthermore, non-dihydropyridine CCBs such as verapamil and diltiazem should generally be avoided in patients receiving VEGF inhibitors because of their potential for cytochrome P450 3A4 drug-drug interactions with anti-VEGF agents [27].

## Autonomic Dysfunction and Blood Pressure Lability in Cancer Survivors

Along with hypertension, autonomic dysfunction is common in both cancer patients and survivors, driven both by treatment-related toxicity and direct effects of malignancy [39]. This often manifests as blood pressure lability, with treatment-induced hypertension, orthostatic hypotension (OH), and baroreceptor injury.

### Orthostatic Hypotension & Labile BP

The presence of cancer is a significant and independent predictor of OH, and people with cancer have a higher prevalence of OH compared to those without cancer (28.7% vs. 16.5%, respectively, *p* = 0.003) [40]. However, cancer survivors experiencing bouts of OH may still be hypertensive at times, as treatment-induced BP lability is a recognized complication of cancer therapy. This may partly reflect the cyclical nature of some cancer therapies, such as sunitinib. Patients receiving this medication can become hypertensive while they are receiving it, then experience significant falls in BP during the off-treatment period [41]. Furthermore, these changes in BP, especially during the off-treatment period, may not be recognized upon BP screening at routine appointments unless patients are monitoring their BP at home as well.

Labile BP in cancer patients is also associated with therapy-related cardiovascular toxicity, highlighting the importance of recognizing and managing these fluctuations. In a study of patients with multiple myeloma receiving proteasome inhibitors, those who developed cardiovascular toxicity related to their cancer therapy had higher baseline BP variability. Systolic BP variability measured during the night-time was independently associated with cancer therapy-related cardiovascular toxicity (adjusted OR 1.09 per unit increase in coefficient of variation; 95% CI 1.01–1.21) [42].

Additionally, the recent AHA statement recognizes the risk of BP lability with VEGF inhibitors and proteasome inhibitors. They recommend at least weekly BP monitoring for the first four to eight weeks on these medications and upon their discontinuation, where rebound hypotension can occur [11].

### Autonomic Dysfunction, Arterial Stiffening, and Baroreflex Injury

A variety of cancers and treatments have been shown to be associated with autonomic dysfunction, which can contribute to labile BP and OH. For example, breast cancer treatment including anthracyclines, trastuzumab, and left-sided radiation is associated with elevated resting heart rate and decreased heart rate recovery after exercise. Furthermore, decreased BP and/or hypotension can be seen in 32–51% of patients receiving these treatments [43]. In the St. Jude Lifetime Cohort study, adult survivors of childhood cancer treated with anthracyclines and/or chest-directed radiation have a two times higher prevalence of autonomic dysfunction and impaired cardiorespiratory fitness [44]. These changes in autonomic function can exacerbate the HTN widely seen in cancer patients and survivors, and they can also be associated with labile BP and orthostatic hypotension (OH), which can complicate treatment options for HTN in cancer patients.

**Fig. 1:**
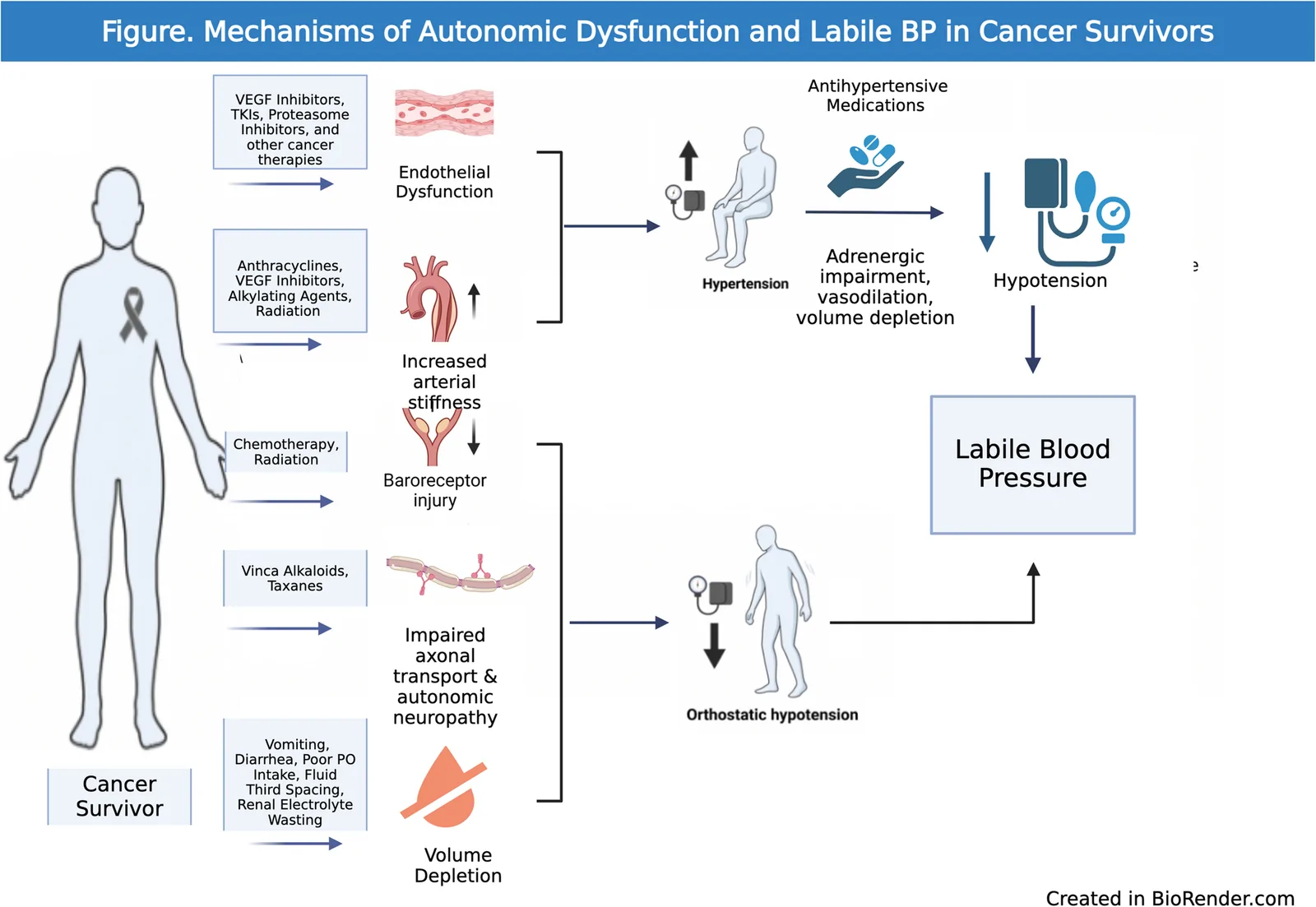
Mechanisms contributing to autonomic dysfunction and labile blood pressure in cancer survivors. Cancer therapies and treatment-related complications can promote hypertension through endothelial dysfunction and increased arterial stiffness, while baroreceptor injury, autonomic neuropathy, and volume depletion can contribute to orthostatic hypotension. Antihypertensive therapy may further exacerbate hypotension, resulting in fluctuations between hypertension and hypotension and contributing to labile blood pressure

Cancer therapies and treatment-related complications can promote hypertension through endothelial dysfunction and increased arterial stiffness, while baroreceptor injury, autonomic neuropathy, and volume depletion can contribute to orthostatic hypotension. Antihypertensive therapy may further exacerbate hypotension, resulting in fluctuations between hypertension and hypotension and contributing to labile blood pressure (Figure 1).

Arterial stiffening is a potential complication of cancer therapy that can attenuate the vasculature’s ability to compensate for changes in positioning, volume status, or vascular tone, which can result in labile blood pressure (Figure 1). Increased arterial stiffening is seen following anticancer therapy in patients receiving anthracycline chemotherapy, VEGF TKIs, and alkylating agents [45]. Patients receiving anthracycline chemotherapy have demonstrated increases in aortic stiffness comparable to 15 years of aging [26]. Increased arterial stiffening is associated with increased incidence of hypertension [46], and in the general population, this also increases the risk of OH by up to 40% [47]. Vascular stiffening could likely be one of the mechanisms associated with the frequent incidence of both hypertension and OH seen in cancer patients. As well as decreasing vascular compliance, vascular stiffening in the general population is also associated with decreased baroreceptor sensitivity [48, 49], which can further hinder the body’s ability to compensate for changes in BP and can result in OH [50].

Interestingly, a new study provides emerging evidence that arterial stiffening during cancer therapy may be preventable in some cases. A secondary analysis of the STOP-CA trial in patients with lymphoma treated with anthracycline chemotherapy found that pretreatment with atorvastatin was associated with preservation of vascular function. Atorvastatin pretreatment was associated with significantly lower odds of an increase (≥ 0.8 m per second) in magnetic resonance imaging–derived aortic arch pulse wave velocity, compared with placebo [51]. This raises the possibility that pretreatment with atorvastatin could mitigate vascular stiffening associated with anthracycline chemotherapy, although it remains unknown whether this could translate into improved BP control.

### Baroreceptor Damage

Baroreceptor damage may occur with cancer treatments through separate mechanisms from arterial stiffening. Radiation, especially for head and neck cancers, is associated with radiation-induced afferent baroreflex failure. Ambulatory BP monitoring of these patients showed a pattern of hypertension, postural hypotension, hypertensive surges, tachycardia, and absence of nocturnal dipping, all in the same patients [52]. Additionally, one study of patients with breast cancer, lymphoma, pancreatic cancer, or prostate cancer, who were all treated with chemotherapy, found that these patients exhibited impaired baroreflex control of arterial BP and increased arterial stiffness relative to non-cancer comparators [53]. This combination of stiffened vasculature and blunted baroreflex sensitivity, along with other contributing factors, may increase the risk of labile BP among cancer survivors (Figure 1).

### Medical Management for Cancer Patients with Orthostatic Hypotension

Although there are no formal guidelines for managing OH in cancer survivors with HTN, treatment recommendations for older adults with HTN and OH have been studied in greater detail. Until additional research and formal guidelines are established for oncology patients, it may be reasonable to utilize treatment recommendations in the general population for some cancer patients. Orthostatic hypotension commonly occurs in older adults, and cancer survivors often experience accelerated biological aging compared to those without cancer [54–56].

Furthermore, the aortic stiffening that occurs after anthracycline chemotherapy is comparable to the vascular changes seen with age [26]. Therefore, although cancer survivors experience unique pathophysiological mechanisms contributing to their OH and autonomic dysfunction, such as baroreceptor injury and volume depletion, some of the vascular changes driving this mechanism may be comparable to those caused by age.

In the general population, a recent scientific statement from the American Heart Association outlines classes of antihypertensive medications most frequently associated with OH in adults with HTN. Because the sympathetic nervous system is involved in maintaining mean arterial pressure, agents that impact the sympathetic nervous system carry the highest risk of OH. This includes peripheral alpha blockers, beta blockers, and central sympatholytics [57]. In individuals without cancer receiving monotherapy for HTN, beta blockers were associated with a twofold and threefold greater risk for developing initial and sustained OH, respectively [58].

Vasodilatory medications, nitrates, phosphodiesterase inhibitors, calcium channel blockers, and diuretics are also associated with increased risk of OH, but these are less implicated with OH than agents acting on the sympathetic nervous system [57]. According to this scientific statement, agents working on the renin, angiotensin, and aldosterone system carry the least risk of causing OH in hypertensive patients [57]. Based on these recommendations in the general population, it is reasonable to avoid beta blockers, alpha blockers, and central sympatholytic agents to treat HTN in cancer patients with OH, and instead consider an agent targeting the renin-angiotensin-aldosterone system. Furthermore, a thorough medication reconciliation should be performed to identify other medications that could cause or exacerbate existing OH. Midodrine and fludrocortisone are commonly used to treat OH, but are not recommended in patients with supine HTN [59].

## Blood Pressure Goals during Cancer Treatment with or without Orthostatic Hypotension

At the present time, there are no specific guidelines for BP targets in patients receiving cancer therapy. Current guidelines from the ACC and AHA suggest blood pressure goals of < 130/80 regardless of additional cardiovascular risk factors [11, 32]. Many large randomized controlled trials investigating HTN in the general population excluded patients with active cancer, which limits the ability to examine treatment goals in cancer patients with hypertension.

### Intensive SBP Control in Cancer Patients and Survivors

However, the recent ECOG-ACRIN trial served as the first randomized controlled trial of BP control in an active cancer population. This trial studied 61 patients with renal cell or thyroid cancer initiating VEGFR-tyrosine kinase inhibitors. They compared intensive SBP control (< 120 mmHg) versus regular SBP control (< 140 mmHg) in patients receiving these anti-VEGF agents. Results from this trial showed that those in the intensive SBP control arm experienced lower average SBP compared to those in the normal control arm (-12.2 (95% CI, -18.1 to -7.0)) mmHg at 1 month, -7.6 (95% CI, -15.3 to -0.4) mmHg at 3 months, and − 6.9 (95% CI, -19.3 to 6.0) mmHg at 6 months. Those in the usual control arm, with SBP goal < 140 mmHg, experienced more adverse events including kidney injury (*n* = 4), hypotension (*n* = 3), and dyspnea (*n* = 2) compared to the intensive control arm. Those with SBP goal < 120 mmHg experienced fewer kidney injuries (*n* = 1), episodes of hypotension (*n* = 2), and no reported dyspnea (*n* = 0) [60].

Although no studies have investigated intensive BP control in oncology patients with HTN and OH, research in the general population suggests that more intensive BP control is associated with a decreased risk of OH as well [57]. Interestingly, these results conflict with those of other studies investigating adverse outcomes in intensive BP control in people with HTN without cancer. One meta-analysis studying intensive versus standard BP control in patients without cancer showed that intensive SBP control (< 130 mmHg) was associated with reduced cardiovascular events, but increased the risk of hypotension, syncope, and acute kidney injury [61]. These conflicting results suggest that intensive BP control in cancer patients may be associated with more favorable outcomes than expected in the general population, but more research is needed to establish this.

Although the SPRINT trial excluded cancer patients, a recent post hoc analysis investigated BP goals in over 2,000 cancer survivors with no active cancer treatment in the past two years. This study found that intensive SBP control (SBP < 120 mmHg) reduced adverse cardiovascular outcomes similarly in both the cancer survivors and non-cancer participants compared to those receiving treatment for standard BP goals (SBP < 140 mmHg) [62]. This suggests that intensive control of SBP may reduce adverse cardiovascular outcomes in patients with a past medical history of cancer. Furthermore, this suggests that a history of cancer, not requiring active treatment in the past two years, should not be a barrier to intensive blood pressure control.

### Suggestions that Intensive SBP Control is Not Contraindicated in Cancer Patients

Overall, the ECOG-ACRIN and SPRINT trials suggest that intensive SBP control (below 120 mmHg) may be feasible for cancer survivors. Intensive SBP control could also be associated with lower risk of cardiovascular events in cancer survivors, decreased risk of kidney injury, hypotension, and dyspnea in those on anti-VEGF agents. However, more research is needed, especially in larger cancer populations receiving a broader variety of anti-cancer therapies, before guidelines can be established for more intensive BP control in cancer patients.

## Lifestyle Factors, Physical Activity, and Cardio-Oncology Rehabilitation among Cancer Survivors with Hypertension

Cardiac rehabilitation and exercise training are shown to significantly reduce BP in hypertensive adults without cancer [63]. Both aerobic and resistance exercise training are shown to reduce systolic and diastolic BP by two to five mmHg in normotensive adults and by five to seven mmHg in hypertensive adults [64]. Exercise training can be as effective as commonly used antihypertensive medications in lowering SBP in patients without cancer [65]. Clearly, exercise is an effective strategy to reduce BP in adults without cancer, and this approach has also been studied in patients with cancer.

A recent trial compared a structured, supervised cardio-oncology rehabilitation program with a community-based exercise intervention in patients with cancer. The cardio-oncology rehabilitation program showed promising BP lowering results compared to the community-based intervention, with a greater mean reduction in systolic blood pressure (− 12.3 [11.8] mmHg vs. − 1.9 [12.9] mmHg; *P* < 0.001), diastolic BP (− 5.0 [5.7] mmHg vs. − 0.5 [7.0] mmHg; *P* = 0.003), and BMI (− 1.2 [0.9] vs. 0.2 [0.7]; *P* < 0.001) [66]. This suggests that exercise is an important and often underutilized approach to controlling HTN in patients with cancer. Furthermore, this reduction in blood pressure is greater than previously documented in a non-cancer population [64], suggesting that exercise training may be especially beneficial in cancer patients.

In addition to improvements in hypertension, exercise programs may also be beneficial for cancer survivors experiencing autonomic dysfunction [67]. Exercise training can also improve cardiac regulation and sympathetic nervous system responses in individuals with breast cancer [68], which could be implicated in both HTN and OH. Although more research is needed to investigate whether exercise can improve OH in cancer survivors, these findings suggest that exercise could be a powerful tool to combat both HTN and autonomic dysfunction.

## Perspectives and Future Directions

Hypertension has emerged as one of the most common and clinically relevant complications encountered in patients receiving treatment for cancer, yet many aspects of antihypertensive therapy are not fully studied. As cancer survival improves, managing cardiovascular disease will become even more important to improve both cardiovascular outcomes and quality of life. Hypertension should be recognized as a dynamic manifestation of cancer biology and treatment-related vascular and autonomic dysfunction.

Further research should focus on:

- Establishing specific blood pressure goals for patients with cancer.
- Comparing antihypertensive therapies across different malignancies and treatment regimens.
- Determining the role of BP lability and autonomic dysfunction in guiding medical management for cancer survivors.
- Investigating the role of nonpharmacologic interventions such as cardio-oncology rehabilitation in treating HTN in cancer survivors.

## Key References

- Cohen JB, Brown NJ, Brown SA, Dent S, van Dorst DCH, Herrmann SM, et al. Cancer therapy-related hypertension: a scientific statement from the American Heart Association. Hypertension 2023;80:e46-e57. doi:10.1161/HYP.0000000000000224.
  - This scientific statement provides a comprehensive framework for the epidemiology, mechanisms, monitoring, and management of hypertension associated with cancer therapies and serves as a foundation for current cardio-oncology hypertension practice.
- Van Dorst DCH, Kabadayi S, Oomen-de Hoop E, Danser AHJ, Mathijssen RHJ, Versmissen J. Treatment and implications of vascular endothelial growth factor inhibitor-induced blood pressure rise: a clinical cohort study. J Am Heart Assoc 2023;12:e028050. doi:10.1161/JAHA.122.028050.
  - This clinical cohort study identifies risk factors for VEGF inhibitor-induced blood pressure elevation and demonstrates that both calcium channel blockers and renin-angiotensin system inhibitors effectively reduce BP during treatment.
- Ganatra S, Barac A, Armenian S, et al. Diagnosis and management of cardiovascular adverse effects of targeted oncology therapies: Bruton’s tyrosine kinase, immune checkpoint, and vascular endothelial growth factor inhibitors: 2025 ACC concise clinical guidance. J Am Coll Cardiol 2026;87:654-82. doi:10.1016/j.jacc.2025.10.018.
  - This recent clinical guidance provides contemporary recommendations for the prevention, monitoring, and management of cardiovascular toxicities associated with targeted cancer therapies, including treatment-related hypertension.
- Frye JN, Sutterfield SL, Caldwell JT, et al. Vascular and autonomic changes in adult cancer patients receiving anticancer chemotherapy. J Appl Physiol 2018;125:198-204. doi:10.1152/japplphysiol.00005.2018.
  - This study identifies vascular and autonomic dysfunction in adults receiving anticancer chemotherapy, supporting the role of impaired vascular and autonomic regulation in the development of blood pressure abnormalities among cancer patients.
- Ky B, Liu T, Margulies KB, Mitchell JD, Haas NB, Gareen IF, et al. Cluster randomized controlled trial of intensive systolic blood pressure control in patients with renal cell or thyroid cancer receiving VEGFR tyrosine kinase inhibitors: ECOG-ACRIN EAQ191. Hypertension 2026;83:e26016. doi:10.1161/HYPERTENSIONAHA.125.26016.
  - This was the first randomized controlled trial evaluating intensive systolic blood pressure control in patients with active cancer receiving VEGFR tyrosine kinase inhibitors and informs the ongoing debate regarding optimal blood pressure targets in this population.
- Li W, Wang Z, Jiang C, Hua C, Tang Y, et al. Effect of intensive blood pressure control on cardiovascular outcomes in cancer survivors. Hypertension 2024;81. doi:10.1161/HYPERTENSIONAHA.123.22194.
  - This study suggests that intensive blood pressure control may reduce cardiovascular events in cancer survivors, supporting consideration of intensive BP management beyond the period of active cancer treatment.
- Viamonte SG, Joaquim AV, Alves AJ, et al. Cardio-oncology rehabilitation for cancer survivors with high cardiovascular risk: a randomized clinical trial. JAMA Cardiol 2023;8:1119-28. doi:10.1001/jamacardio.2023.3558.
  - This randomized clinical trial demonstrates the potential of structured cardio-oncology rehabilitation to improve blood pressure and cardiovascular risk factors in cancer survivors, supporting exercise as an important component of hypertension management.

## Data Availability

No datasets were generated or analysed during the current study.
